# Spatial and temporal analysis of liver cancer mortality in Yunnan province, China, 2015–2019

**DOI:** 10.3389/fpubh.2022.1010752

**Published:** 2022-09-27

**Authors:** Chengcheng Feng, Jinghua Liu, Hailiang Ran, Linxiong Wu, Xuemeng Liang, Hao Sun, Yuanyuan Xiao, Wei Chang

**Affiliations:** ^1^School of Public Health, Kunming Medical University, Kunming, China; ^2^Department of Gastroenterology, Affiliated Hospital of Yunnan University, Kunming, China

**Keywords:** liver cancer, mortality, spatial autocorrelation, temporal trend, time-series data

## Abstract

Liver cancer is a major public health challenge. Few published studies reported temporal trend and geographical distribution of liver cancer mortality in China, especially in less developed southwest regions with higher liver cancer incidence. In the current study, we obtained liver cancer mortality data from population-based death surveillance system in Yunnan province in 2015–2019. The mortality of liver cancer was analyzed by using the joinpoint regression model. The space distribution of liver cancer mortality in 129 counties and districts in Yunnan province was illustrated by using the ArcGIS software. Moran's I method was used to estimate the global and local spatial autocorrelation of liver cancer mortality. Analytical results revealed that from 2015 to 2019, the average mortality rate of liver cancer in Yunnan province was 12.96/100,000, with an average annual growth rate of 6.26% (*p* < 0.05). Higher liver cancer mortality was found in rural areas and in males. Moreover, people aged 45–50 years experienced a steep increase in liver cancer mortality rate. High-high cluster was mainly consisted of areas with higher hepatitis virus infection rate or severe intravenous drug use problem. Our study results suggest a heavy burden of liver cancer in southwest China Yunnan province. Comprehensive intervention measures need to be developed and implemented.

## Introduction

Cancer is a major health threat. New cancer cases and cancer related deaths had been estimated around 19.3 and 10 million in 2020 (1). Liver cancer is one of the most common carcinomas. In 2020, with over 0.9 million new cases and over 0.8 million deaths, liver cancer ranked 6th and 3rd by incidence and mortality among all cancers globally (2). East and southeast Asia, north and west Africa report higher incidence and mortality of liver cancer (3–5). Although China has made great efforts in the past decades, liver cancer remains one of the malignant tumors with the highest incidence and mortality today (6). In China, liver cancer accounted for 45.27 and 47.12% of new cancer cases and cancer related deaths in 2020 (1). Geographically, southwest regions of China report higher prevalence of liver cancer (7).

The majority of existing studies have identified risk factors of liver cancer at individual level, such as age, sex, socioeconomic status, overweight, hepatitis B virus (HBV) or hepatitis C virus (HCV) infection, excessive alcohol consumption and smoking (8–10). Although they are valuable in guiding primary intervention policies and measures of liver cancer, for effective regional prevention and control, an extra endeavor needs to be done to disclose spatial and temporal characteristics of liver cancer, to help determine high risk areas of intervention priority. At present, spatial epidemiology has gradually been used in describing geographical distribution of cancers in China. For instance, recently, a spatial and temporal study of gastric cancer indicated that the mortality of gastric cancer varied between different regions of China, and environmental management was probably behind this discrepancy (11). Another Chinese study explored spatial and temporal pattern of lung cancer in Shenzhen (12).

However, few studies had been published regarding to spatial and temporal patterns of liver cancer mortality in China (13–15). Moreover, these studies only briefly described time or space distribution of liver cancer mortality at the country level or in some developed coastal areas, no published study has ever elaborately discussed this issue in impoverished inland provinces, which reported much higher liver cancer incidence and disease burden. In the current study, aiming to address this deficiency, we investigated spatial and temporal distributions of liver cancer mortality in southwest China Yunnan province from 2015 to 2019.

## Materials and methods

### Data sources

Liver cancer cause specific deaths in Yunnan province at county level in 2015–2019 were obtained from the all-cause death registration system which incorporated into the China Disease Prevention and Control Information System. In this population-based death registration system, cause of death is coded according to the International Classification of Diseases (ICD-10). In this study we only extracted deaths caused by liver cancer (coded as C22.0-C22.9). Population data at county level of Yunnan province were obtained from the resident population information module which also incorporated into the China Disease Prevention and Control Information System, and the age standardized mortality rates were calculated by using the 2010 Chinese standard population: we divided age into 18 groups from 0 to 85 (5 years a group), and then used the direct standardization method to calculate the age standardized mortality rate (ASR) by using the formula $ASR=\frac{\overset{A}{\underset{i=1}{\sum }}a_{i}w_{i}}{\overset{A}{\underset{i=1}{\sum }}w_{i}}\times 100,000$ (i: age group i; a_i_: observed mortality rate in age group i; w_i_: the standard population size in age group i). The digital maps of Yunnan (illustrating at county level) were freely obtained from the National Geomatics Center of China (https://www.webmap.cn/main.do?method=index, accessed on 16/3/2022) and the official Planning Cloud website (http://www.guihuayun.com/, accessed on 16/3/2022).

### Statistical analysis

#### Joinpoint regression analysis

The joinpoint regression, also known as piecewise linear regression, is an ideal tool for analyzing time series data (16). In some previously published studies, this method had been successfully used to assess changing trends in cancer mortality (17, 18). Here in this study, we used joinpoint regression model to calculate the average annual percentage change (AAPC) of liver cancer mortality in Yunnan province in 2015–2019: when AAPC > 0, it indicates an increasing trend of liver cancer mortality; when AAPC < 0, it indicates a decreasing trend of liver cancer mortality; when AAPC = 0, it indicates no change (19).

#### Spatial distribution analysis

Liver cancer mortality rates in Yunnan province were linked to the digital map of Yunnan province by using a primary geographic key at county level (altogether 129 counties). In order to make the map uniform across years, all counties were divided into six groups based on their liver cancer mortality rates (in units of 7/100,000) and differentiated by colors.

#### Spatial aggregation analysis

The global and local spatial autocorrelation analyses were further performed. At first, Moran's I was used as an indicator to reflect global spatial aggregation in the whole region and significance test was performed. When the Moran's I value > 0, it indicates a positive spatial autocorrelation; when the Moran's I value < 0, it indicates a negative spatial autocorrelation; when the Moran's I value = 0, it indicates a random distribution (20). The LISA plots were then used to measure local spatial autocorrelation of liver cancer mortality in Yunnan Province. When the local Moran's I was significant, the LISA plot can reflect four types of spatial aggregation: high-high, where high value regions are adjacent to regions with high values; high-low, where high value regions are adjacent to regions with low values; low-high, where low value regions are adjacent to regions with high values; and low-low, where low value regions are adjacent to regions with low values.

#### Softwares

Data merging and sorting were performed by using the Stata/SE software (Version: 16.0). Joinpoint regression analysis of liver cancer mortality was performed by the Joinpoint software (Version: 4.9.0.0). Regional distribution of liver cancer mortality was mapped by using the ArcGIS software (Version: 10.8.1). The GeoDa software (Version: 1.18.0) was used for global and spatial autocorrelation analysis. Significance level for all statistical tests or inferences was set as a two-tailed probability no higher than 0.05.

## Results

### General characteristics of liver cancer mortality in Yunnan

The total, sex-specific, and region-specific mortality of liver cancer in Yunnan province from 2015 to 2019 are reported in Table 1. During the study period, a total of 30,921 liver cancer deaths were reported, with an overall crude mortality rate of 12.96/100,000 (calculated as: $\frac{average\;deaths}{average\;population\;size}\times 100,000$) and an age standardized rate (ASR) of 13.62/100,000. There were 22,813 deaths in males and 8,099 deaths in females, with a much higher ASR in males (20.29/100,000) than in females (6.93/100,000). Liver cancer deaths in rural and urban regions were 22,776 and 8,136, with a higher ASR in rural region (15.47/100,000) than in urban region (10.21/100,000).

**Table 1:** Liver cancer mortality rates by sex and region in Yunnan, China, 2015–2019.

**Year**	**Indicators**	**Sex**		**Region**		**Total**
		**Male**	**Female**	**Urban**	**Rural**	
2015	Deaths	3,793	1,479	1,493	3,779	5,272
	Population size	24,193,816	22,947,571	16,455,390	30,685,997	47,141,387
	Crude mortality rate (1/100,000)	15.68	6.45	9.07	12.32	11.18
	ASR (1/100,000)	18.07	6.76	9.90	13.80	12.40
2016	Deaths	4,259	1,459	1,637	4,081	5,718
	Population size	24,587,267	22,830,733	16,598,539	30,819,461	47,418,000
	Crude mortality rate (1/100,000)	17.32	6.39	9.86	13.24	12.06
	ASR (1/100,000)	19.29	6.38	10.33	14.30	12.87
2017	Deaths	4,824	1,636	1,660	4,800	6,460
	Population size	24,546,762	23,163,240	16,696,929	31,013,073	47,710,002
	Crude mortality rate (1/100,000)	19.65	7.06	9.94	15.48	13.54
	ASR (1/100,000)	22.31	7.19	10.60	17.03	14.72
2018	Deaths	4,808	1,730	1,612	4,926	6,538
	Population size	24,909,028	23,095,950	16,927,521	31,256,857	48,004,978
	Crude mortality rate (1/100,000)	19.30	7.49	9.52	15.76	13.62
	ASR (1/100,000)	21.25	7.41	9.85	16.87	14.39
2019	Deaths	5,129	1,795	1,734	5,190	6,924
	Population size	25,057,937	23,241,970	15,493,370	32,806,537	48,299,907
	Crude mortality rate (1/100,000)	20.47	7.72	11.19	15.82	14.34
	ASR (1/100,000)	21.09	7.14	10.79	15.74	14.10
2015–2019	Average deaths	4562.60	1619.80	1627.20	4555.20	6182.40
	Average population size	24658962.00	23055892.80	16434349.80	31316385.00	47714854.80
	Average mortality rate (1/100,000)	18.50	7.03	9.90	14.55	12.96
	ASR (1/100,000)	20.29	6.93	10.21	15.47	13.62

ASR, age standardized mortality rate by using the Chinese population in 2010.

### Trends of liver cancer mortality

**Figure 1:**
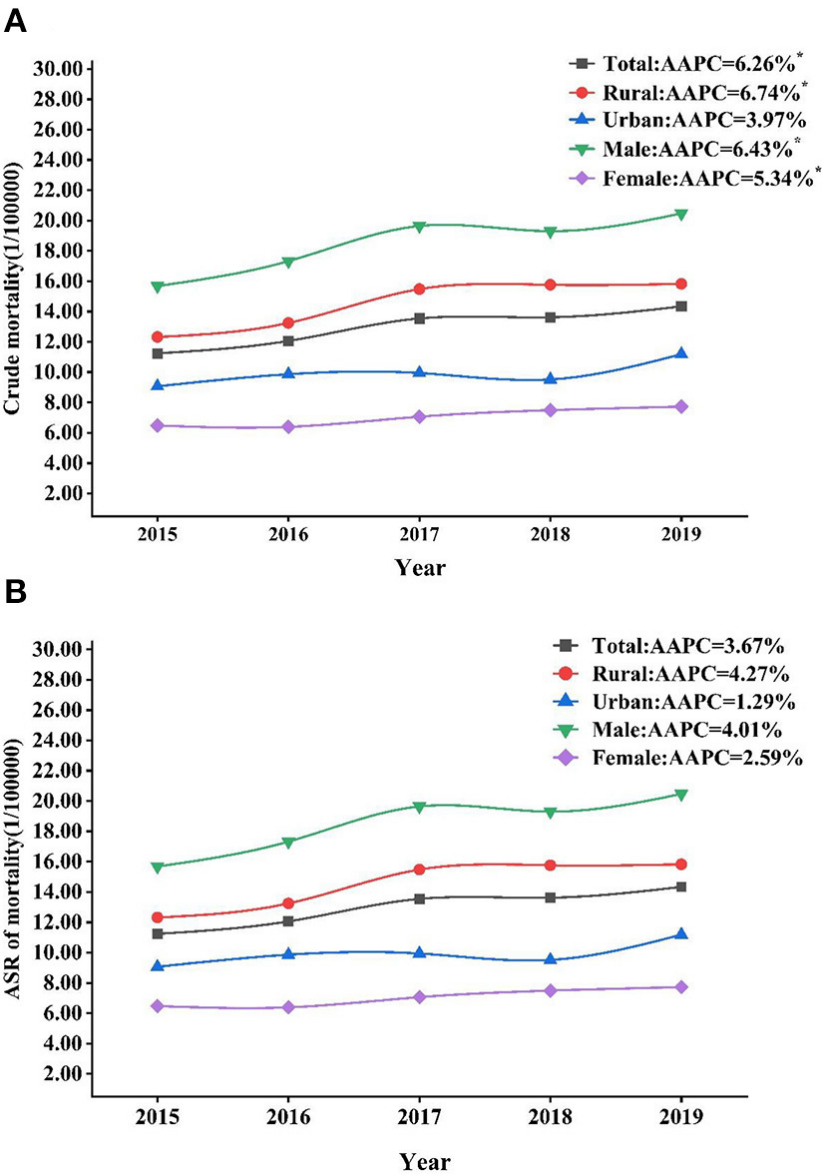
Trends of crude mortality rates **(A)** and ASRs **(B)** of liver cancer in Yunnan by sex and region, 2015–2019. ASR, age standardized rate. *p < 0.05.

The joinpoint regression results indicated that there was no statistical significance in the time point segmentation, suggesting that the trend and degree of changes in liver cancer mortality rate by sex and region were in general similar from 2015 to 2019. Crude mortality rates of liver cancer in males, females, and rural region all showed an increasing trend from 2015 to 2019 (total AAPC: 6.26%, male AAPC: 6.43%, female AAPC: 5.34%, rural AAPC: 6.74%, all with *p* < 0.05). However, from 2015 to 2019, there were no significant trends for the ASRs of liver cancer mortality in Yunnan (Figure 1).

**Figure 2:**
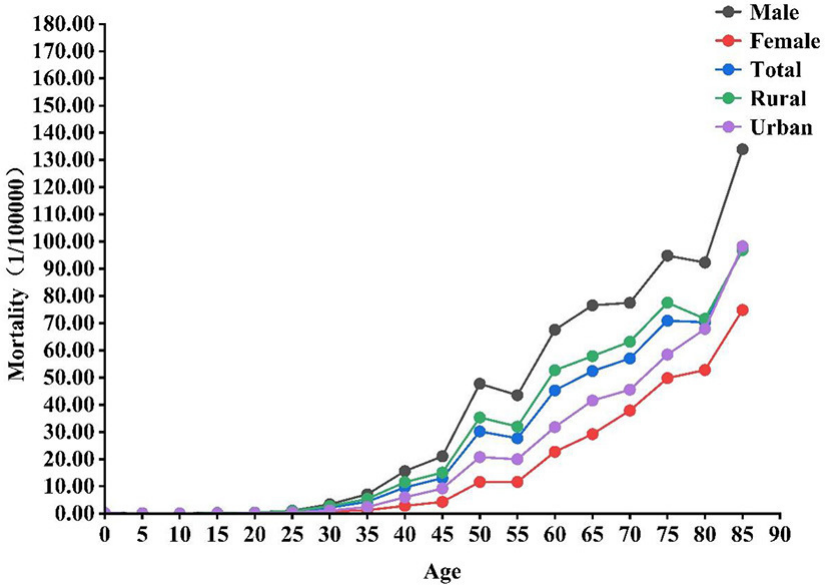
Age-specific mortality rates of liver cancer by sex and region in Yunnan, 2015–2019.

For all liver cancer deaths in Yunnan province in 2015–2019, the average age at death was higher for females (66.29 ± 13.83 years) than for males (60.15 ± 13.44 years), higher for urban residents (63.31 ± 13.86 years) than for rural residents (61.21 ± 13.75 years). The mortality rate of liver cancer gradually increased from the age of 30 years onwards, and the 80–85 and 45–50 age groups were seen the most prominent increase. The overall trends in age-specific mortality rates for liver cancer by sex and region were generally similar (Figure 2).

### Spatial distribution of liver cancer mortality

**Figure 3:**
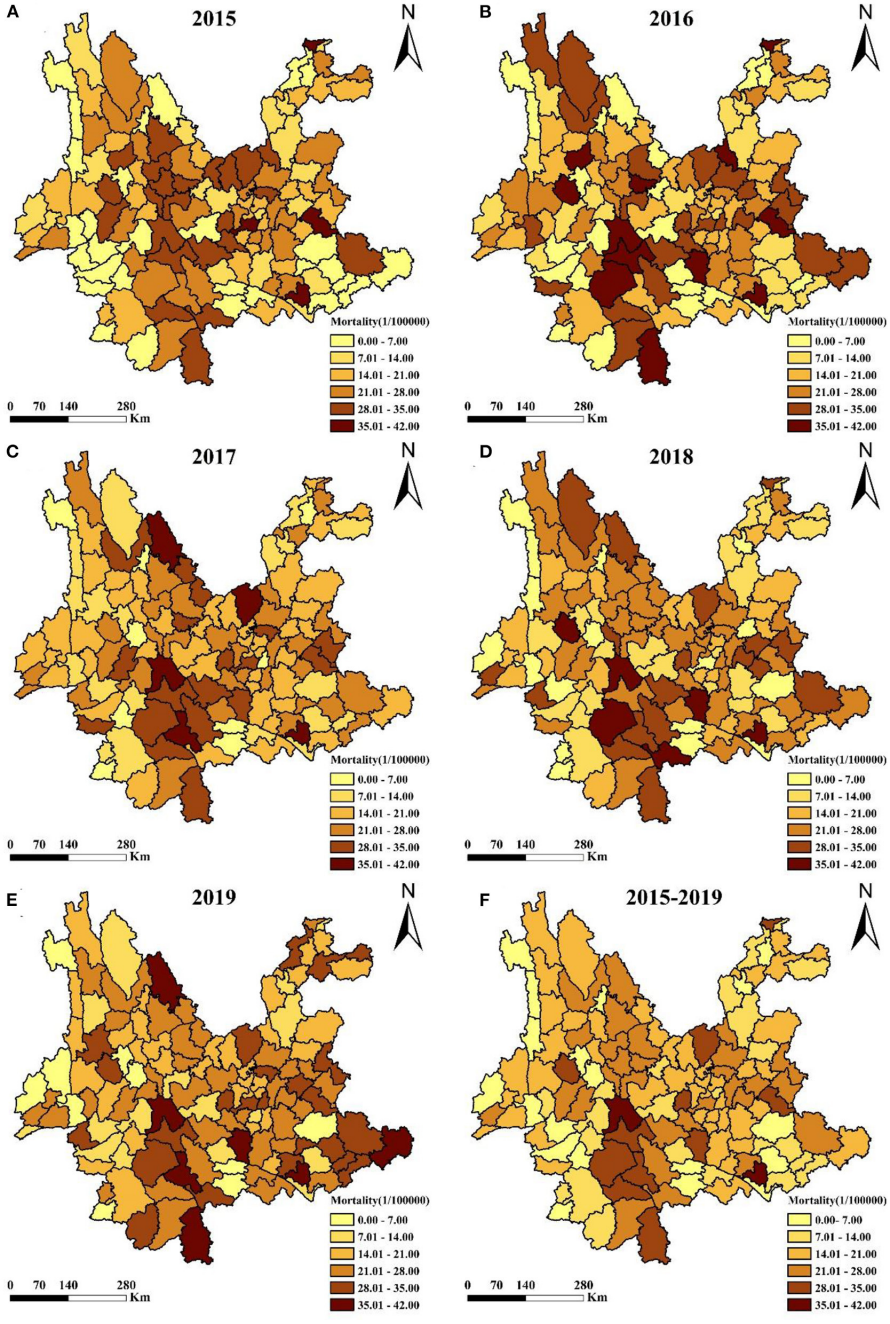
Geographic distribution of liver cancer mortality rates at county level in Yunnan, 2015–2019. **(A–E)** year 2015–2019, **(F)** year 2015–2019 on average.

According to the distribution maps, liver cancer mortality was generally at a higher level from 2015 to 2019 in Pingbian, Jingdong, Mengla, Shizong and Shiping counties, with Pingbian county in the south consistently led liver cancer death rate. Liver cancer mortality in most counties fluctuated between 2015 and 2019, whereas continuously increased in Yingjiang, Qubei, Dongchuan, Huize, and Jiangchuan counties, continuously decreased in Yongshan, Nyingchi, Yuanyang, Yanshan, Xichuan, and Menghai counties (Figure 3).

### Spatial aggregation of liver cancer mortality

**Figure 4:**
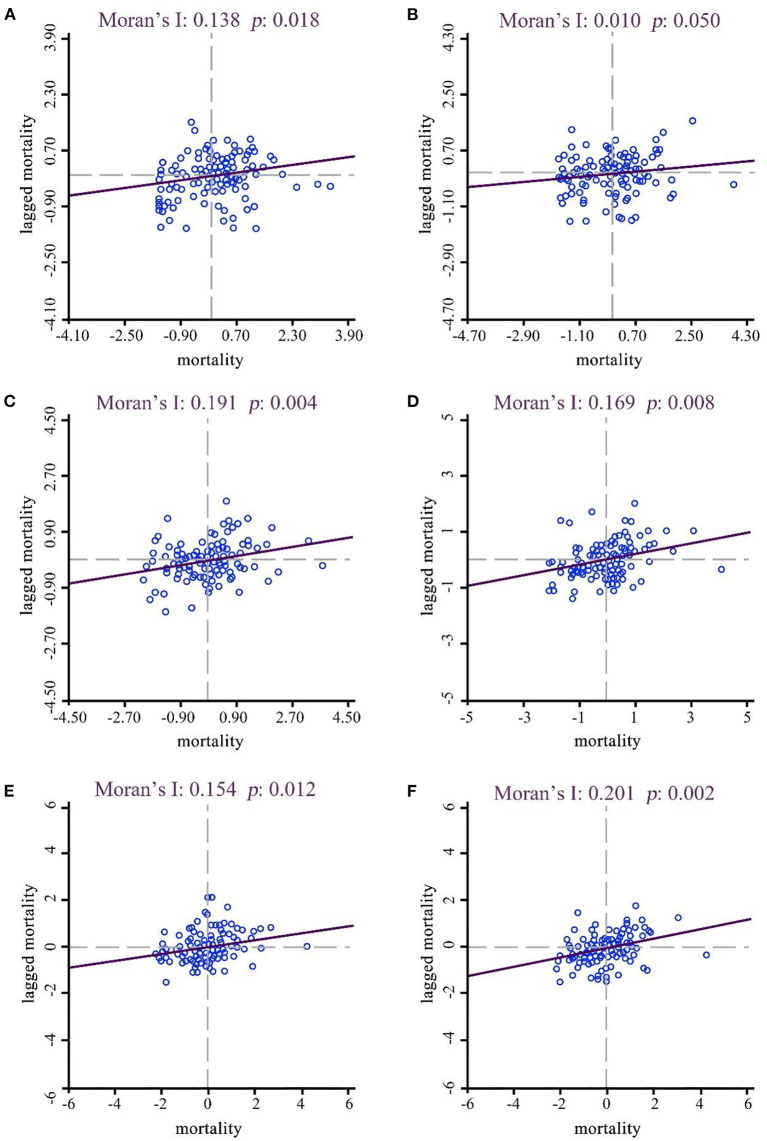
Moran's I scatter plots of liver cancer mortality in Yunnan, 2015–2019. **(A–E)** year 2015–2019, **(F)** year 2015–2019 on average.

**Figure 5:**
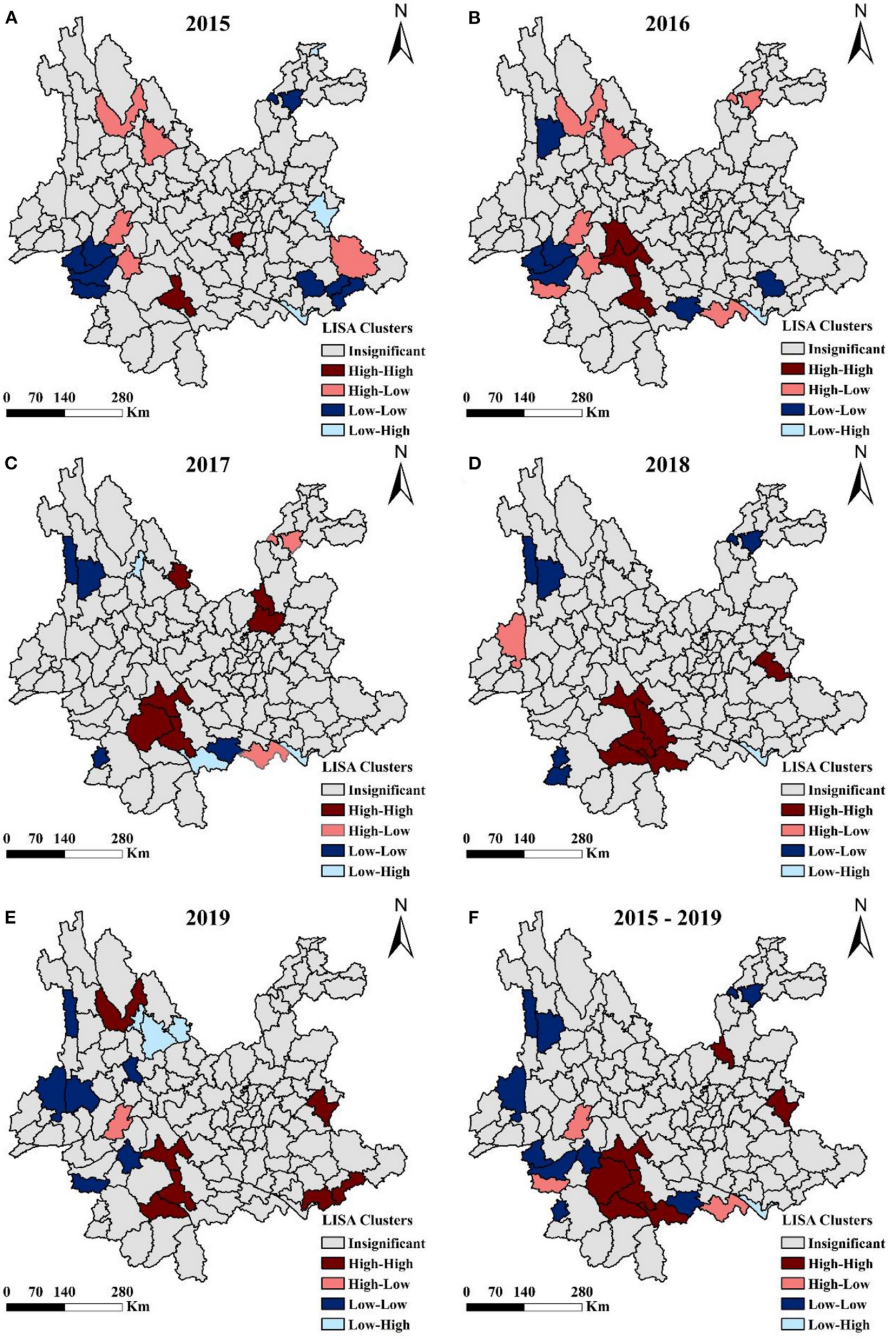
LISA maps of liver cancer mortality in Yunnan at county level, 2015–2019. **(A–E)** year 2015–2019, **(F)** year 2015–2019 on average.

The global and local autocorrelation analyses of liver cancer mortality in Yunnan province in 2015–2019 were shown in Figure 4. The global autocorrelation results revealed that the Moran's I values for the year 2015 to 2019 were 0.138, 0.101, 0.191, 0.169, and 0.154, respectively, suggesting that liver cancer mortality in Yunnan province was spatially positively correlated across regions in 2015–2019 (Table 2). Further local autocorrelation analysis results were presented in Figure 5: LISA plots revealed high-high cluster in southwest regions of Yunnan, like Ninger, Jingdong and Zhenyuan counties, low-low cluster in western border areas. All clustering types in the graphs were statistically significant.

**Table 2:** Global spatial autocorrelation of liver cancer mortality in Yunnan, 2015–2019.

**Year**	**Moran's I**	***Z*-value**	***P*-value**	**E[I]**	**Mean**	**SD**
2015	0.138	2.10	0.018	−0.008	−0.012	0.071
2016	0.101	1.62	0.050	−0.008	−0.011	0.069
2017	0.192	2.93	0.004	−0.008	−0.010	0.069
2018	0.169	2.62	0.008	−0.007	−0.011	0.068
2019	0.154	2.29	0.012	−0.008	−0.005	0.069
Total	0.201	3.13	0.002	−0.008	−0.008	0.067

## Discussion

In this study, we performed spatial and temporal analysis for liver cancer mortality in Yunnan province in 2015–2019. Based on analytical results, we have the following major findings: first, the overall mortality of liver cancer in Yunnan province was at a high level and still presented a slow increase; second, the mortality of liver cancer varied considerably by different characteristics of the population, such as region, sex and age; third, spatial autocorrelation analysis identified some high-high areas in liver cancer mortality, which should be prioritized for liver cancer prevention and control.

The age standardized mortality rate of liver cancer in Yunnan province was 14.10/100,000 in the year 2019, much higher than the national average rate of 8.44/100,000 (21), reflecting a higher level of liver cancer burden in Yunnan. This high liver cancer mortality probably can be attributed to HBV/HCV infection, as HBV infection rate was much higher in Yunnan (49.92/100,000) when compared with other regions in China, like Jiangsu province (16.17/100,000) and Shanghai (35.07/100,000) (22). Besides, Yunnan province is also an important epidemic center for blood-borne viruses such as HCV (23, 24). Meanwhile, Yunnan province is an economically underdeveloped region locates in southwest border of China. It has been reported that the participation rate in cancer screening was only about 50% in Yunnan (25, 26), which may further contribute to increased liver cancer incidence and mortality due to inefficient primary and secondary prevention measures caused by socioeconomic predicaments. The slow increase in liver cancer mortality in Yunnan province between 2015 and 2019 that we observed in this study was consistent with the national trend (27). This increased liver cancer mortality probably was the consequence of elevated HBV prevalence from 36.17/100,000 in 2004 to 49.92/100,000 in 2015 in Yunnan province (22).

Regarding to regional distribution, we found that liver cancer mortality was significantly higher in rural areas than in urban areas, and this difference was consistent with findings in other Chinese provinces (14, 28). Urban-rural differences in risk factors (HBV infection, aflatoxin intake) exposure, socioeconomic development, hygiene status, and accessibility to health care (including treatment accessibility) may collectively contribute to this discrepancy in liver cancer mortality (29, 30). Based on gender, a higher liver cancer mortality had been found in males, also consistent with the findings of previously published Chinese studies (31, 32). Males reported higher levels of exposure to some identified behavioral risk factors of liver cancer, such as smoking and alcohol abuse (33, 34). Besides, sex related steroid hormones, immune responses, and epigenetic differences may also play a role (35, 36). For age distribution, an interesting finding to be noticed is that the 45–50 age group was seen a steep increase in liver cancer mortality. A study from Shanghai also reported a sharp increase in liver cancer mortality in residents aged above 40, and concluded that the high mortality in this age group probably can be attributed to HBV infection (37). One study showed that HBV infection rate in Chinese residents aged 40–59 was 181.62/100,000, significantly lower than which in the younger age groups (22). This phenomenon could be the consequence of the fact that HBV vaccine was not introduced into China until the 1980s (38, 39).

Spatial autocorrelation analysis revealed that liver cancer mortality in Yunnan was in general not randomly distributed: Dongchuan district and Xundian county in Kunming, Ning'er county, Jingdong county, Zhenyuan county, Jiangcheng county in Pu'er city, and Malipo county in Wenshan city, were all seen high liver cancer mortality rates. It is well-known that HBV/HCV infection is the most important risk factor of liver cancer in China (40). Reports indicated that Kunming city had the highest HCV infection rate (15.26/100,000) in Yunnan in 1990–2014, and Pu'er city had the highest HBV infection rate (66.19/100,000) in 2015 (41, 42). Therefore, areas reported high liver cancer mortality rates in this study were largely regions with high HBV/HCV infection, suggesting that liver cancer mortality is closely related to local hepatitis epidemic (41). Moreover, Yunnan province also reports a higher prevalence of HIV infection caused by injecting drugs, due to its geographical location (next to the Golden Triangle). Yingjiang county, where the liver cancer mortality rate was also high, is the same county that mostly affected by illicit drug use and AIDS epidemic in Yunnan (43). Previous studies have shown that HIV infection caused by injecting drugs was more likely to be co-infected with HBV or HCV (44, 45).

The high-high cluster of liver cancer mortality in Yunnan province, as disclosed by autocorrelation analysis, mainly includes areas with high hepatitis virus infection rate or widespread intravenous drug abuse, and these areas are undoubtedly of the highest priority for liver cancer prevention and control. A comprehensive intervention strategy which incorporates multiple effective measures from different aspects should be considered. For instance, more health care resources need to be allocated to these areas, to help increase HBV/HCV vaccination rate and improve treatment quality of hepatitis patients. Moreover, for drug users, local health departments should provide effective counseling and referral services, promote methadone maintenance treatment clinic initiatives (46–48). However, some high-high clustering areas, like Malipo, Yuanjiang and Luoping, are neither hepatitis epidemic nor drug abuse areas, the primary risk factors that are driving up local liver cancer mortality should be further investigated, preferably by using case-control studies.

Our study has several limitations. First, restricted by data availability, we only analyzed liver cancer mortality in 5 years, therefore the long-term liver cancer mortality trend in Yunnan province needs to be continuously updated, to help build an ideal prediction model. Besides, the spatial and temporal distributions of liver cancer incidence are also of considerable study interest, which should be further investigated in the future. Second, the accuracy of study results could be influenced by the quality of surveillance data. Finally, we only preliminarily analyzed spatial and temporal trends of liver cancer mortality, in subsequent studies, some important influencing factors, especially environmental factors, could be further included.

## Conclusion

In this study, by using spatial and temporal analysis, we found a high liver cancer mortality rate with increasing trend in southwest China Yunnan province from 2015 to 2019, especially in rural areas and in males. Moreover, a sharp increase has been identified for residents aged 45–50. The high-high cluster in liver cancer mortality largely includes areas with high local HBV/HCV infection rate or severe intravenous drug abuse problem. Comprehensive intervention measures should be adopted in reducing liver cancer burden in Yunnan. Future studies with updated surveillance data and include important environmental covariates are warranted.

## Data availability statement

The raw data supporting the conclusions of this article will be made available by the authors, without undue reservation.

## Author contributions

YX and WC designed the study and critically revised the manuscript. CF, JL, HR, LW, XL, and HS carried out the data collection. CF and HR performed data analysis. CF and JL prepared the draft manuscript. All authors critically revised the manuscript for important intellectual content, contributed to the article, and approved the submitted version.

## Funding

The study was supported by Joint Special Project of Yunnan Provincial Department of Science and Technology, Kunming Medical University for Applied Basic Research [No. 2019fe001(-178)], and Top Young Talents of Yunnan Ten Thousand Talents Plan [No. YNWR-QNBJ-2018-286].

## Conflict of interest

The authors declare that the research was conducted in the absence of any commercial or financial relationships that could be construed as a potential conflict of interest.

## Publisher's note

All claims expressed in this article are solely those of the authors and do not necessarily represent those of their affiliated organizations, or those of the publisher, the editors and the reviewers. Any product that may be evaluated in this article, or claim that may be made by its manufacturer, is not guaranteed or endorsed by the publisher.
